# [Corrigendum] Identification of lncRNA EGOT as a tumor suppressor in renal cell carcinoma

**DOI:** 10.3892/mmr.2024.13194

**Published:** 2024-03-06

**Authors:** Lu Jin, Jing Quan, Xiang Pan, Tao He, Jia Hu, Yifan Li, Yaoting Gui, Shangqi Yang, Xiangming Mao, Yun Chen, Yongqing Lai

Mol Med Rep 16: 7072–7079, 2017; DOI: 10.3892/mmr.2017.7470

Subsequently to the publication of the above paper, an interested reader drew to the authors’ attention that, concerrning the Transwell cell migration and invasion assay data shown in Fig. 6A and B for the 786-O cell line on p. 7206, the pcDNA3.1-EGOT ‘Migration’ and ‘Invasion’ (a-1 and b-1) data panels appeared to contain overlapping sections of data, such that they were potentially derived from the same original source, where these panels were intended to show the results from differently performed experiments. The authors have re-examined their original data, and realize that the ‘Invasion’ (b-1) panel in Fig. 6B was inadvertently chosen incorrectly.

The revised version of Fig. 6, now featuring the correct data for the ‘Invasion’ experiment (B1 in the replacement figure) in Fig. 6B, is shown on the next page. Note that this error did not adversely affect either the results or the overall conclusions reported in this study. All the authors agree with the publication of this corrigendum, and are grateful to the Editor of *Molecular Medicine Reports* for allowing them the opportunity to publish this. They also wish to apologize to the readership of the Journal for any inconvenience caused.

## Figures and Tables

**Figure 6.**
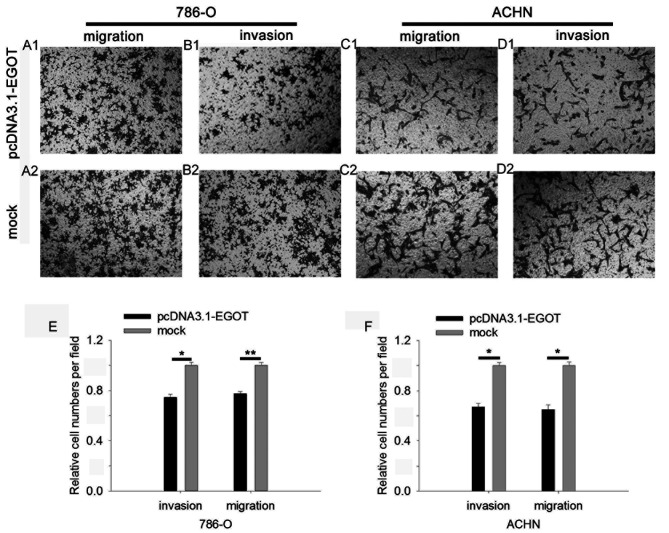
Overexpression of EGOT inhibits RCC cell migration and invasion. Representative images are presented of (A) the migration of 786-O cells, (B) the invasion of 786-O cells, (C) the migration of 786-O cells and (D) the invasion of ACHN cells. Quantification of the invasion and migration of (E) 786-O cells and (F) ACHN cells is displayed. *P<0.05 and **P<0.01. EGOT, eosinophil granule ontogeny transcript; RCC, renal cell carcinoma..

